# She'll Be ‘Right… but Are They? An Australian Perspective on Women in High Performance Sport Coaching

**DOI:** 10.3389/fspor.2022.848735

**Published:** 2022-06-17

**Authors:** Alexandra H. Roberts, Anthea Clarke, Caitlin Fox-Harding, Georgia Askew, Clare MacMahon, Sophia Nimphius

**Affiliations:** ^1^Sport and Exercise Science, La Trobe University, Bundoora, VIC, Australia; ^2^School of Medical and Health Sciences, Edith Cowan University, Joondalup, WA, Australia; ^3^School of Exercise and Nutrition Sciences, Queensland University of Technology, Brisbane, QLD, Australia

**Keywords:** women, coaching, sport, representation, sport leadership, female athletes, diversity and inclusion, gender equity

## Abstract

Participation and media coverage of women in high-performance sport has been steadily increasing in recent years throughout the world. While this increase in interest has led to many young women and girls becoming involved in grassroots sport, there has yet to be a significant change in the number of women in coaching roles, particularly at the high-performance level. This paper synthesizes and summarizes the current challenges facing women sport coaches in Australia, drawing from existing research, media and government reports to understand the barriers for women entering and progressing in these roles. We also present some of the more recent initiatives to increase opportunities for women in high performance coaching. Within Australia, there is a need to (1) understand the pipeline for women coaches, (2) examine the interacting contexts and constraints that women are subject to within sporting organizations, and (3) create a preliminary framework for future research, outreach, and education to address gender inequity within Australian sport coaching.

## Introduction

Participation of women^1^ in high-performance sport has been steadily increasing in Australia and internationally, with the last 10 years frequently referred to as the decade that saw the rise of women's sport throughout the world (Konstantopoulos, 2019; Mitchell, 2019; Thompson, 2020). This “fitful but undeniable progress” (Clarey, 2018) is due, in part, to multiple factors that contribute to the overall professionalization of women in sports, such as increased investment, increased broadcasting and subsequent revenue, and increased awareness of and demand for women in sport from the public (Taylor, 2020). Progress is also potentially due in part to deliberate government policies and investments focused on improving women's participation (e.g., the Victorian Government's “Change Our Game” grant scheme; Casey et al., 2019). Research conducted by information and data measurement company *Nielsen* suggests that women in sport have a broader engagement, influence, and value than most realize (Perry, 2019) and consulting company *Deloitte* predicts that women's sports will be a billion-dollar industry in the near future due to their “mass-market appeal” (Lee et al., 2020). These “bright spots” (Lebel et al., 2021) demonstrate the growing potential for equity in high-performance sport, and provide evidence that deliberate strategies for diversity are having tangible results.

While it is important to acknowledge the growth in women's divisions of sport, referring only to the athletes ignores the many roles women can play in women's and men's sports. According to former Sport Australia CEO Kate Palmer, there is an important distinction to be made between “women's sport” and “women in sport” (Palmer, 2019). Current research regarding “women in sport” focuses on increasing participation or performance of women athletes in different sports (Sharrow, 2017; Cooky and Antunovic, 2020; Slaten et al., 2020; Eime et al., 2021b). However, research focusing on women in “supporting” or “servicing” roles (including, but not limited to, coaches, officials, sport science/sports medicine, and administrators) has been missing in the quantity of research and actual representation (Moore et al., 2001; Reade et al., 2009; Courtney et al., 2020; Wicker and Kerwin, 2020). One of the most considerable and visible discrepancies between “women's sport” and “women in sport” is the lack of women in coaching roles. There is a further lack of clear female pathways to establish a pipeline from grass roots to high performance coaching. While this lack of representation is not limited to Australia, the cultural dominance of sport and the high participation rates in Australia are unique (ABC News, 2016; Department of Health, 2018). These unique cultural factors mean it is important to examine the current numbers, environment and potential opportunities and barriers faced by women coaches in Australia. Given this position, and the need for broader socio-cultural exploration, this article provides an overview of the current limited knowledge of women coaches in Australia and demonstrates the need for further research in this space.

## Participation of Women Coaches

Despite the proportion of Australian women athletes at the 2016 Rio Olympic Games being 50.6%, and higher than men for the first time in Australian history, the number of non-athlete women that were provided accreditations for the games (i.e., officials or coaches) declined to 9% (down from 12% in the 2012 London Olympic Games) (Norman, 2014; ABC News, 2016; Sport AUS, 2017; Ward, 2020). Australia's numbers have yet to be released for the Tokyo 2020 games but are likely to be similar given that the International Olympic Committee has reported that just 13% of coaches at the delayed Games were women, despite women making up 49% of competitors (International Olympic Committee [IOC], 2021). Similarly, when professional Australian team sports are examined, there is a dearth of women in coaching roles, for either women's or men's teams. As of the 2021–22 season, in the 14-team Australian Football League Women's competition (AFLW), there are currently no women in senior coaching roles, and in the six-year history of the competition, there have been only three women who held these roles (in comparison to 23 men in the same period) (Black, 2021a). In the same time-frame, the Women's National Basketball League (WNBL) have two women head coaches out of eight teams (25%), while the A-League Women's football (soccer) competition currently have three women head coaches across nine teams (33%). There are no women coaches in any Australian professional men's teams, nor in the Australian National teams of soccer, cricket, or rugby union. This is in line with international trends, where there is almost a total absence of women coaching male athletes (LaVoi, 2019). However, it is not inconceivable to have women in coaching roles in professional men's sport, with the 2021 seasons for NFL (12 women) and MLB (23 coaches) showing that the introduction of women coaches can be done so effectively (Genet, 2021; Iannaconi, 2021).

Even when women coaches are successful at the highest level of their sport, they can be met with derogatory and detrimental backlash (including sexist and homophobic behavior) and struggle to gain or maintain employment at the highest levels of coaching. For example, Bec Goddard, who has coached two professional teams in two different codes (AFLW and WNBL) to championships, struggled to find full-time employment as a coach in any league, with any gender, despite her track record of success (Halloran, 2021). Similarly, Lisa Alexander, head coach of the Australian Diamonds netball team for nine years with an 81% win record, has been very vocal around her disappointment at the “old boys' network” that prevented her from even being interviewed for a coaching position in Australian Football (AAP, 2016). Describing the sport as a “closed shop for women,” Alexander has emphasized that good coaching transcends sport, and that her gender should not dictate which sports and levels she is capable of coaching (Halloran, 2021). Even with these relatively high-profile women coaches speaking out about the difficulties they face in the coaching sphere, women's experiences in coaching are often not well-documented. Exploring the knowledge, practices and experiences of both current coaches and those who have left the profession are necessary first steps toward dismantling the many systemic barriers that face women coaches in Australia.

There is limited information available regarding women coaches at the Australian sub-elite and community levels, with an unacceptable lack of understanding of women's experiences at these levels (Hotham et al., 2021). In 2013, the Australian Bureau of Statistics published a report on “Women in Sport,” which determined that over 680,000 Australian women are involved in sport in a “non-playing” capacity (often in addition to being a player). However, these numbers do not differentiate between the many non-playing roles required for sport to operate, and count coaches along with officials, team managers, and operational roles such as merchandising and canteen duties. A 2021 paper from Eime et al. (2021a) and colleagues found that between 2016 and 2018 there was a 6% increase in the number of women coaching in Australia, however there was no capacity to differentiate which level/s these women coached at. Understanding how many women are in coaching roles at all levels of Australian sport is an integral first step in finding ways to support the development and retention of these coaches specific to their needs. Further, understanding the factors that promote and, conversely, prevent women from entering or staying in the coaching pipeline is essential to increasing participation and retention rates across the spectrum from community-level, volunteer coaches to elite professional and high-performance coaches.

## Benefits of Women as Coaches

The coaching process is described in empirical research as complex, contextual, dynamic, social, and pedagogical (Townsend et al., 2015; Stodter and Cushion, 2019). Across all levels of sport, coaches play an integral role in the development of athletes' skills and performance capabilities (Mallett and Dickens, 2009; Nash and Sproule, 2009; Dehghansai et al., 2019). Coaches also play other influential roles, including mentor, parent, friend, teacher, role model, psychologist, or negotiator (Côté et al., 2007). However, in almost every sport globally, the proportion of coaches who are women is a “statistical minority” (Marshall, 2010; Acosta and Carpenter, 2012).

There is significant evidence that women want, need and benefit significantly from having women role models in all areas of development, including sport. Moreover, it is not only women who benefit from having women in coaching roles (LaVoi, 2016). Outside of sport, there is substantial emerging evidence that companies with gender-diverse leadership perform better than their male-dominated counterparts in nearly all aspects of performance–they have been shown to be more innovative, generate more revenue and profits, have a broader customer base, occupy a greater market share, and have more people vying for positions within the organization (Moreno-Gómez et al., 2017; Lafuente and Vaillant, 2019; Fine et al., 2020).

Conversely, a lack of women in leadership can lead to many unfavorable outcomes for women and girls who participate in sport, including (but not limited to) a devaluation of their abilities, a loss of self-confidence, and the inability to challenge or resist negative gender stereotyping (LaVoi and Dutove, 2012; Perry, 2019). Further, the lack of women in coaching reduces the opportunity to disrupt the “other” discourse (as described by De Haan and Knoppers, 2020), and from this discourse a perpetuation of the marginalization of women athletes and coaches will (inevitably) remain (de Haan and Sotiriadou, 2019). Women athletes coached by men are also less likely to pursue a career in coaching (or other sport-related careers) compared to those who were coached by women (LaVoi and Dutove, 2012; Wells and Hancock, 2017; Pike et al., 2018; Sotiriadou and de Haan, 2019). The advantages of having more women as coaches benefits not only the individual and their athletes, but clubs, organizations and, more broadly, society.

## Initiatives to Increase the Participation of Women in Coaching

With the recognized need for better representation and inclusion of women in sport (Allison and Barranco, 2020; Gaston et al., 2020; Love et al., 2020), there is still limited scientific evidence and support of specific approaches to ensure sustainable development for women in coaching. In recent years, several organizations have been formed to empower women coaches through advocacy, education, and promotion of women in coaching at all levels [such as the Women's Coaching Association (AUS) and the Female Coaching Network (UK)]. Common initiatives that are implemented in an attempt to increase the participation of women coaches include women-only development and accreditation courses, targeted recruitment of current and former women athletes, and increasing the profile of existing women coaches (Women in Sport, 2015; Women's Coaching Association, 2021). Within Australia, there are several women-focused high-performance coaching development and accreditation programs such as the women-only cohort of the Australian Institute of Sport's Elevate Coach Program (Australian Institute of Sport, 2021) and the Women's Coaching Academy for Australian football coaches (Black, 2021b). These programs, which also target ex-athletes and have a strong media presence to raise the profile of the coaches involved, demonstrate the growing commitment of sporting organizations to develop women coaches at the high-performance level. These frequent, but often temporary, initiatives demonstrate a rising institutional awareness of the need for more women in high-performance coaching roles, but the efficacy of these programs and whether they lead to a sustained increase of women in high-performance coaching remains to be seen.

In addition to development, it is important to consider the recruitment and progression of women coaches. There are many barriers to women entering the coaching profession, and equally as many for retention and progression after entry. To the best of the authors' knowledge, there is no initiative within Australia (or internationally) that systematically cultivates and transitions coaches from grassroots to high-performance coaching; rather, “survival of the fittest” determines which coaches make this transition. Women face socio-cultural pressures to act in a “caregiving” or “nurturing” role and as a result are often pigeonholed into coaching only junior athletes, or face discrimination in organizational settings due to the lack of family-friendly policies, forcing women to choose between their family and child-rearing responsibilities and their coaching role (LaVoi and Dutove, 2012). These ecological barriers see women being overlooked for promotion to higher-level coaching roles and make it difficult for women to progress or return to the coaching workforce after having children. Little research has been conducted to identify key support areas to encourage the continued involvement and promotion of women coaches, at organization, cultural, or individual levels.

## Future Directions

- The purpose of this piece was not to provide new information, but rather to demonstrate the lack of knowledge about the experiences of women coaches in Australia and to discuss the benefits inherent within increasing the number and quality of women high-performance coaches. In line with this purpose, we propose a number of future directions that researchers or sporting organizations may wish to explore in recruiting, developing and retaining Australian women high-performance coaches.
- Mixed-method studies to enable both tracking and measurement of the numbers of women in different levels of coaching, as well as understanding women's experiences in recruitment, development and retention at different levels (e.g., community vs. elite), employment status (volunteer vs. full time) and in different sports.
- Use of theoretical frameworks (e.g., organizational change theory or social-ecological systems) to examine and compare the systemic, organizational and policy barriers and challenges faced by women in different sports.
- Research engagement and collaboration with systems at local, state and federal levels to seek funding for research on industry-based, practical topics with real-world program outcomes and a-priori establishment of implementation plans.
- Creation of coaching networks for both women and allies.
- Creation of “report card” frameworks for sporting organizations (similar to Athena Swan) that rates sports and organizations on their commitment to and capacity for supporting women coaches at different levels.
- Deliberate strategies to promote the visibility of women in coaching roles.
- Similar research agendas could be formed in relation to women in other sporting roles, as these questions, issues and future directions are relevant for all roles that women play in the sporting industry.

## Conclusion

There are clear benefits in developing women coaches and a need for more research to focus on the unique contributions that women coaches can bring to both men's and women's sport at all levels of the pathway. Despite increasing calls to include and promote women, it is currently unknown how many Australian women are coaching at any level, which makes it difficult to pinpoint the reasons for a lack of women in high-performance roles. While the concepts discussed in this paper are similar to many Western countries, Australia's unique cultural relationship with sport and interest in maintaining an international reputation for sporting excellence brings with it a need to examine the Australian-specific context of women coaches. Therefore, to improve our understanding of the complex issue within Australian sport of improving the representation of women in high-performance coaching in Australia, extensions on international research focused on women athletes (de Haan and Sotiriadou, 2019) using a multi-level theoretical lens (Burton and Leberman, 2017) can be applied, distancing prior focus on individual factors and extending the analysis to organizational and socio-cultural factors. Alternatively, frameworks to support gender equality in other sectors (e.g., the Athena Swan Charter found in higher education; Advance HE, 2020) could be used to monitor conditions and outcomes for women working as sport coaches in Australia while providing specific guidance for organizations. Research into the roles and experiences of women in other Western countries, particularly the UK, USA, Canada and New Zealand can be used as a springboard for research and discussion.

This paper sought to summarize the current state of Australian women coaches and demonstrated that there is a significant gap in this space, not the least of which is a lack of understanding of the current numbers and roles of women coaches. Future research must first understand the current context, including the numbers, roles, and levels of women coaches in Australia, before then seeking to understand the context-specific barriers and facilitators that women face in their progression in a coaching career. Change in understanding the participation of women in high performance sport coaching will not result from the efforts of any one single entity or organization; but rather requires a multifaceted, united approach. A key consideration within a collaborative approach to change is the need for capacity and supportive environments that will allow women coaches to thrive. While numerous approaches to improving the participation of women coaches have been completed in the past and in other countries, critical examination of these initiatives and policies are required if we are to gain true insight and understanding and elicit real change within Australian high-performance coaching.

## Data Availability Statement

The original contributions presented in the study are included in the article/supplementary material, further inquiries can be directed to the corresponding author.

## Author Contributions

AR, AC, CF-H, GA, CM, and SN contributed to all stages of the conceptualization and design of the work. AR, CF-H, and GA drafted the initial version of this perspective piece. All authors contributed to the final production, approved the final version to be published, and agreed to be accountable for all aspects of the work.

## Funding

This work was funded in part through a Research Focus Area grant in 2019 from La Trobe University.

## Conflict of Interest

AR and SN have separate collaborations with advocacy groups relating to the content of the article. All authors are involved with national sporting organizations from an employment or volunteer perspective.

## Publisher's Note

All claims expressed in this article are solely those of the authors and do not necessarily represent those of their affiliated organizations, or those of the publisher, the editors and the reviewers. Any product that may be evaluated in this article, or claim that may be made by its manufacturer, is not guaranteed or endorsed by the publisher.

## Footnotes

^1^The authors have followed the United Nations guidelines for gender-inclusive language. Gender is a social construct comprised of roles, behaviours, activities, and attributes within a given society at a certain time, whereas sex refers to biological characteristics of living beings. Therefore, the words “wo/men” and “wo/man” are used instead of “fe/male.” Further, the pronouns used reflect pronouns used in each respective report.
